# Cusp fusion pattern in bicuspid aortic valve disease predicts severity of aortic flow abnormalities

**DOI:** 10.1186/1532-429X-15-S1-O69

**Published:** 2013-01-30

**Authors:** Malenka M Bissell, Aaron T Hess, Steffan J Glaze, Alex Pitcher, Matthew D Robson, Alex J Barker, Saul Myerson, Stefan Neubauer

**Affiliations:** 1 Oxford Centre for Clinical Magnetic Resonance Research, University of Oxford, Oxford, UK; 2 Departments of Radiology and Biomedical Engineering, Northwestern University Feinberg School of Medicine, Chicago, IL, USA

## Background

Bicuspid aortic valve disease (BAV) is associated with aortic dilation. We examined whether cusp fusion pattern altered the severity of aortic flow abnormalities and ascending aortic wall shear stress (WSS).

## Methods

We prospectively enrolled 80 patients (51 with right-left-coronary-cusp fusion pattern [RL-BAV] and 29 with right-non-coronary-cusp fusion pattern [RN-BAV]), and 27 healthy volunteers (HV) with a mean age across groups of 42±16 years. Time-resolved 3-dimensional flow-sensitive magnetic resonance imaging was used to quantify flow abnormalities and local distribution of WSS in the ascending aorta. The amount of helical blood flow was quantified using the planar fluid circulation measure. ‘Normal' flow patterns in HV involved a very mild right or left-handed helix (mean planar fluid circulation 3.2± 2SD: -5 to +11 mm^2^/s). Abnormal right-handed helical flow pattern was defined as planar fluid circulation >11 mm^2^/s and abnormal left-handed helical flow pattern as <-5 mm^2^/s. Complex flow was visually defined as disintegration of the helical flow pattern.

## Results

Both BAV groups showed predominantly abnormal flow, but with significant differences. In the RL-BAV group, 10% had normal flow patterns, 80% had abnormal right-handed flow and 10% had complex flow. There was no left-handed flow. The RN-BAV group showed more severe flow abnormalities in general, with no normal flow, a mainly right-handed flow pattern in 66%, complex flow in 24% and left-handed flow in 10%. Circumferentially averaged systolic WSS (WSSsyst) increased with the severity of helical flow abnormality: 0.58±0.20 N/m^2^ vs. 0.83±0.27 N/m^2^ vs. 1.15±0.35 N/m^2^ in normal, right-handed and left-handed flow respectively. WSSsyst in complex flow patterns was lower than helical patterns (0.72±0.26 N/m^2^), implying that the lack of organised helix in complex flow reduces the WSS, but still not to normal.

To determine the impact of cusp fusion pattern on aortic size, flow disturbance and WSS independent of the flow pattern, we compared BAV patients with the commonest flow pattern (right-handed flow). RN-BAV had larger ascending aortic diameters adjusted for body surface area (1.95 vs. 1.75 cm/m^2^, p=0.04) and higher planar fluid circulation value (36.5 vs. 26.6 mm2/s, p=0.009) compared to RL-BAV. Both subgroups had similar WSSsyst, high anterior total WSS values and normal posterior total WSS. Splitting the total WSS into its components, RN-BAV had a higher in-plane WSS (0.62 vs 0.45 N/m^2^, p=0.006).

## Conclusions

RN-BAV showed more severe flow abnormalities and larger ascending aortas. These findings are the first potentially causative indication that cusp fusion pattern is important in determining adverse aortic outcome.

## Funding

This study is funded by the British Heart Foundation.

**Figure 1:**
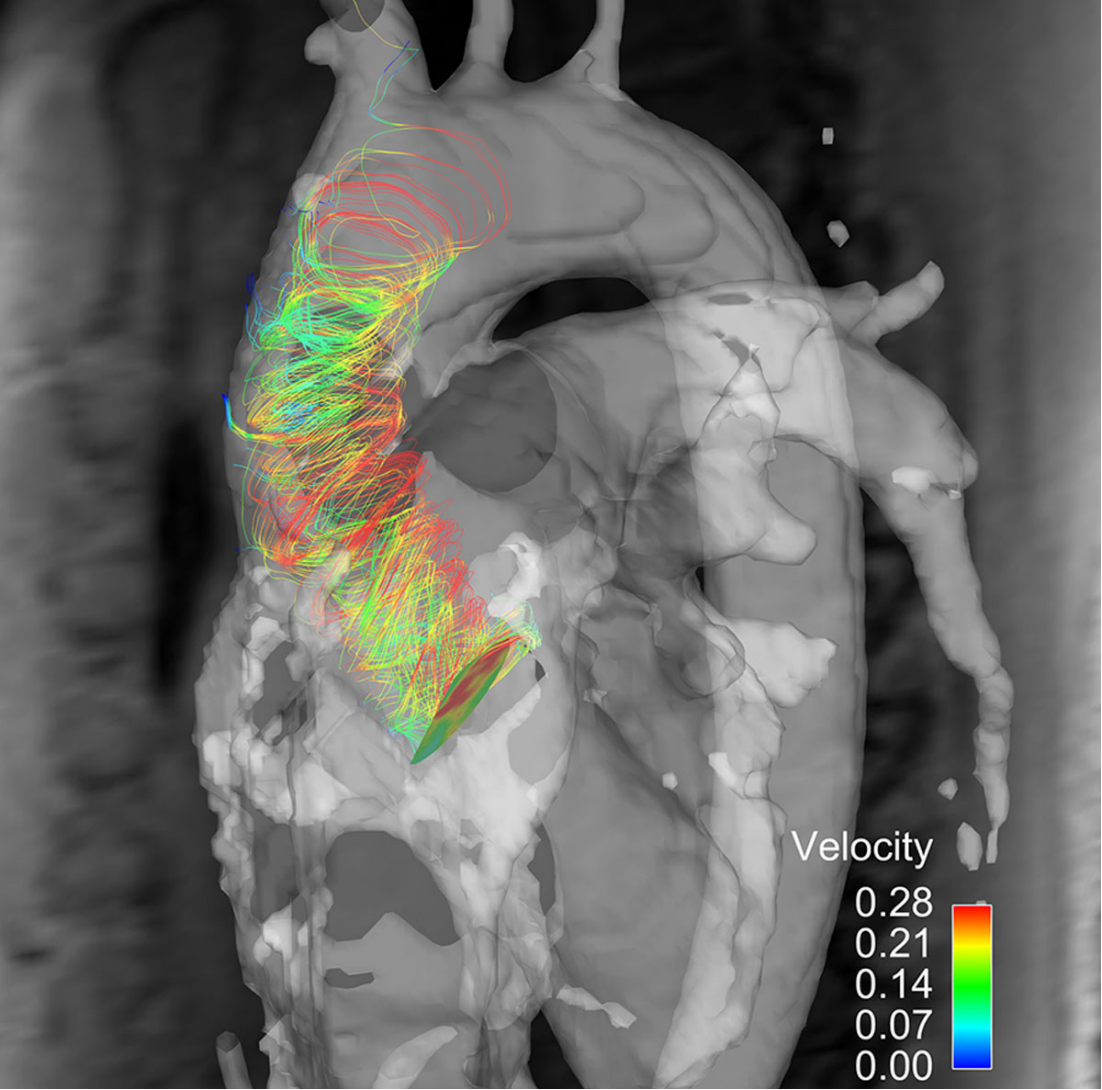
Right-handed helical flow pattern in bicuspid aortic valve disease

**Figure 2:**
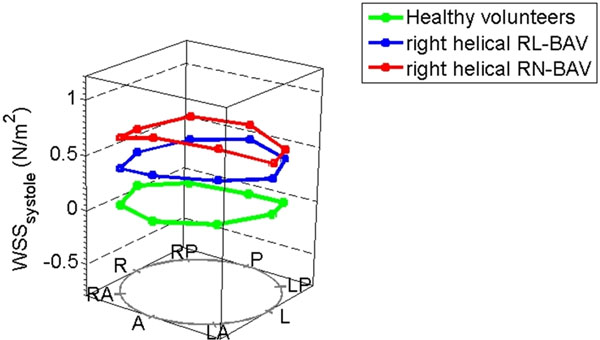
In-plane wall shear stress patterns in the ascending aorta

